# Wunderlich Syndrome: A Seldom Presentation of a Relatively Ubiquitous Tumor

**DOI:** 10.7759/cureus.56126

**Published:** 2024-03-13

**Authors:** Sarthak Sharma, Iqbal Ali, Varun Shetty

**Affiliations:** 1 Department of General Surgery, Dr. D.Y. Patil Medical College Hospital and Research Centre, Dr. D.Y. Patil Vidyapeeth, Pune, Pune, IND

**Keywords:** angiography, angioembolization, nephrectomy, angiomylipoma, wunderlich syndrome

## Abstract

Wunderlich syndrome (WS) is characterized by spontaneous renal or perinephric hemorrhage in the absence of known trauma. WS is much rarer than haemorrhage caused by iatrogenic or traumatic conditions. The classic WS presentation of Lenk's triad of acute onset flank pain, flank mass, and hypovolemic shock is seen in less than a quarter of patients. The majority of patients present with only isolated flank pain and are frequently imaged in the emergency department with an unenhanced computed tomography (CT). The underlying aetiology varies, with the majority of cases attributed to neoplasms, vascular disease, cystic renal disease, and anticoagulation. We hereby present a case of an 80-year-old female who presented with severe discomfort in her left flank for three days in the absence of trauma. The patient was brought in a state of hypovolemic shock. After a thorough evaluation, a diagnosis of WS secondary to angiomyolipoma was made, for which selective angioembolization of the left renal artery was performed. However, due to further deterioration, a left nephrectomy had to be performed. The patient had an uneventful recovery and showed no deterioration on follow-up. The treatment modalities are vivid depending on the hemodynamic status of the patient, ranging from conservative management to operative intervention, and should be tailored to the vital state on admission.

## Introduction

Wunderlich syndrome (WS) represents spontaneous renal or perinephric bleeding that takes place without any external trauma [1]. A rare but severe condition, WS may be managed conservatively with the exception of hemodynamically unstable pts warranting surgical intervention. Although most patients report non-specific symptoms, such as nausea and vomiting, much graver consequences like life-threatening hemorrhage have also been reported. Lenk’s triad encompasses sudden onset flank pain, a palpable flank mass, and hypovolemic shock, which constitutes the classic presentation of WS. Up to 60% cases are neoplastic in origin, for example, angiomyolipoma and renal cell carcinoma. The probability of tumor rupture resulting in potentially fatal internal hemorrhage increases when the size of angiomyolipoma exceeds 40 mm in diameter [2].

Over time, the advent of interventional radiology techniques has provided surgeons with a new weapon in the scarce armada against WS. We hereby aim to throw light on a similar case that we encountered and tackled in a step-up approach.

## Case presentation

An 80-year-old female visited our surgical OPD with severe discomfort in her left flank for three days. She had a history of multiple episodes of bilious vomiting for the past three days. The patient did not give any history of trauma, hematuria, haematochezia, hematemesis, and anticoagulation. She did provide with history of a weight loss of ~10 kg in the past six months. Her clinical examination elicited a tender, bimanually palpable lump in the left lumbar region with localized guarding. On presentation, she had severe hypotension and metabolic acidosis and was shifted to the surgical ICU for resuscitation. She was intubated due to respiratory distress and was maintaining a steady blood pressure on ionotropic support. Blood work revealed a hemoglobin of 6 g/dl along with elevated acute phase reactants, such as CRP. Arterial blood gas revealed severe metabolic acidosis with an elevated lactate level.

The remaining blood parameters were unremarkable. Ultrasound of the abdomen showed free fluid in the flanks and pelvis with an edematous left kidney. Post-stabilization of the patient, a contrast study of the abdomen demonstrated a large left angiomyolipoma with a large perinephric hematoma(400-450 cc), causing anterior displacement of the left kidney extending inferiorly in the retroperitoneum.

**Figure 1:**
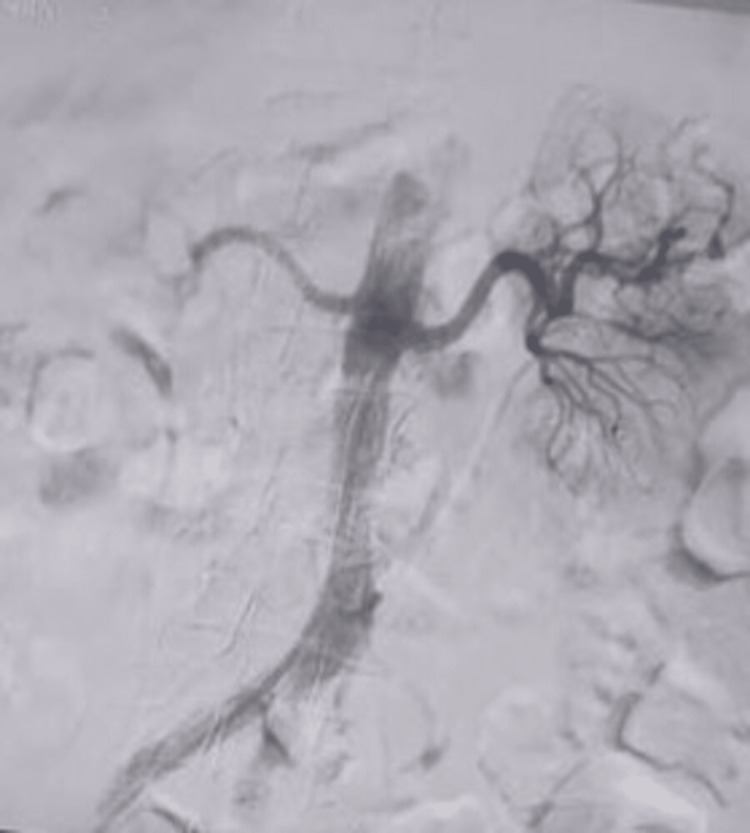
Left renal artery embolization using coils: angioembolization photograph

On serial evaluation using six hourly Hb levels and hemodynamic status, the patient did not show any improvement, and ionotropic support had to be escalated. Considering her hemodynamic instability, she was subjected to left renal artery embolization in the interventional radiology (IR) suite (Figure 1). Post-procedure, the patient showed only limited improvement, but the patient continued to be on a higher ionotropic setting.

**Figure 2:**
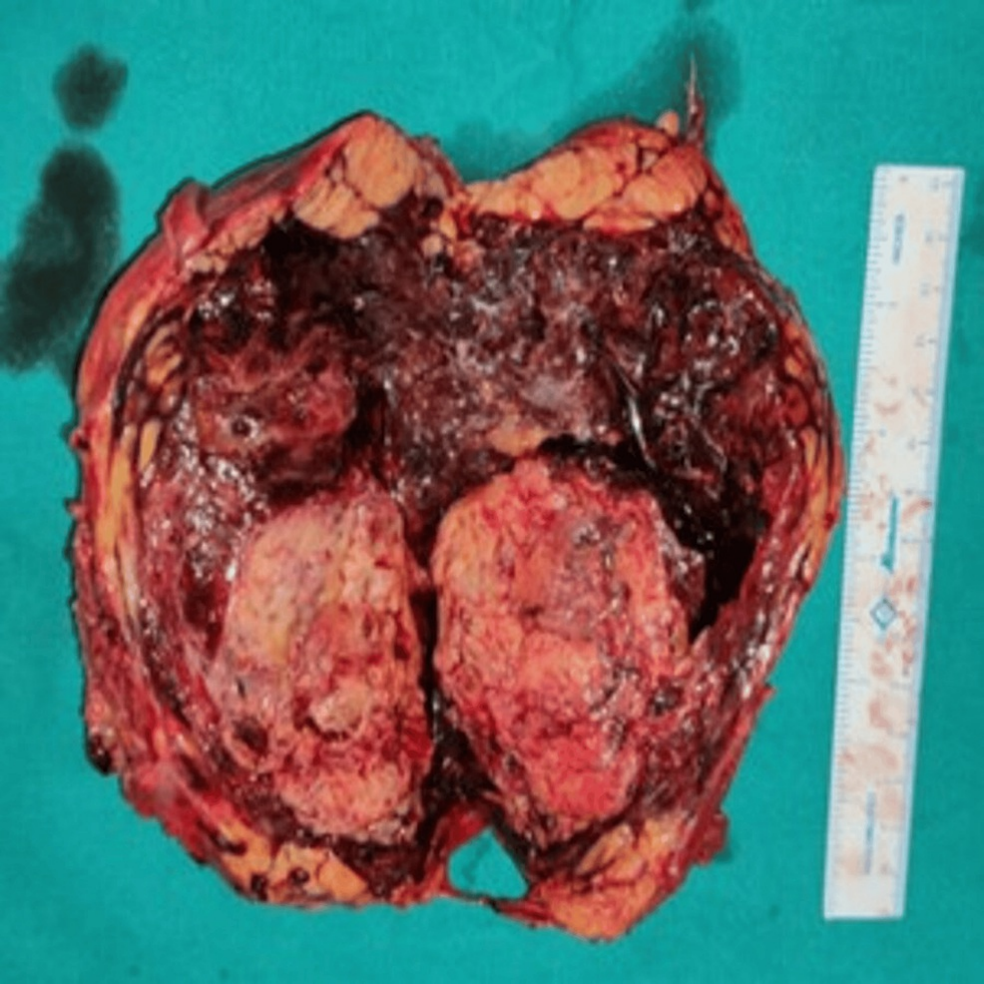
Left nephrectomy specimen with angiomyolipoma involving the lower pole of the kidney and a large perinephric hematoma.

We decided to proceed with surgical intervention 24 hours after embolization because all other options had been exhausted. On exploration, a large left renal angiomyolipoma with a perinephric hematoma was identified, and subsequently, a left nephrectomy was performed (Figure 2).

The procedure was uneventful, and the patient had a smooth postoperative recovery with weaning off ionotropic support and return to the ward after three days. The final histopathology report was consistent with a benign angiomyolipoma with hemorrhage. On the review visit after three months, the patient was symptom-free, and follow-up blood work showed a drastic improvement in the previously deranged parameters.

## Discussion

In the realm of clinical practice, it has been observed that spontaneous renal hemorrhage (SRH) is an infrequent occurrence accompanied by significant adverse consequences. In the context of the lack of antithrombotic medication and absence of trauma, the condition manifests as intraparenchymal renal hemorrhage [1]. The most prevalent aetiologies of SRH include vascular malformations; vasculitides, such as polyarteritis nodosa; and hidden vascular renal tumors, such as renal cell carcinoma or angiomyolipoma. Renal neoplasms are the predominant etiological factor in the occurrence of SRH, constituting approximately 65% of all reported cases [3-5].

The occurrence of the Lenk's triad has been observed in patients with retroperitoneal hemorrhage, such as in those with SRH. This triad typically presents as the progressive start of flank pain, the presence of a palpable flank mass, and the development of hypovolemic shock.

The diagnosis of SRH might be complicated because of its capacity to imitate specific acute abdominal diseases, such as aortic dissection. SRH is often incidentally detected through the utilization of ultrasonography or contrast-enhanced computed tomography (CT) imaging techniques. Ultrasound serves as a valuable tool for expeditiously detecting the problem; however, it is imperative to corroborate its findings with a CT scan, which additionally elucidates the underlying cause of the hemorrhage. Several publications recommend obtaining an angiography in cases where the CT reporting is not helpful in determining the underlying etiology. If radiological investigations fail to identify the underlying cause, it is recommended to proceed with surgical exploration and biopsy [6,7].

There exist many management recommendations that are contingent upon the occurrence of active bleeding and the overall hemodynamic condition of the patient. The initial surgical findings indicated that immediate nephrectomy was recommended for all cases. Nevertheless, new research indicates that it may be prudent to use a conservative approach using various therapeutic measures, such as hydration, pain management, blood product replacement, and intravenous fluid administration for volume resuscitation [8-10]. In cases where there is suspicion of an infectious cause, it is advisable to initiate adequate antibiotic medication. Selective artery embolization is considered a viable approach for managing active bleeding, and numerous experts advocate for its use as the definitive treatment. Nevertheless, nephrectomy remains the primary method of choice. Therefore, therapeutic advice frequently align with the author's personal experience.

## Conclusions

Among the plethora of causes responsible for left flank pain with shock, WS, although rare, needs due consideration while ruling out the differentials. Despite the scarcity of records available on this presentation, the significance of prompt and appropriate management cannot be overstated. A relatively benign tumor-like angiolipoma although usually indolent may present as WS, which has a rougher course. The treatment modalities are vivid depending on the hemodynamic status of the patient, ranging from conservative management to operative intervention. By diving into this rare presentation and its characteristics and clinical outcomes, we aim to shed light on the complexities and subtleties of a dreaded complication, a relatively benign condition that can rise.
